# Analysis of the mediating effects of self-efficacy and self-control between physical activity and Internet addiction among Chinese college students

**DOI:** 10.3389/fpsyg.2022.1002830

**Published:** 2022-09-23

**Authors:** Zhihao Du, Xiuli Zhang

**Affiliations:** ^1^School of Physical Education and Sports, Beijing Normal University, Beijing, China; ^2^Research Center for Sports-Medicine Integration Development, Zhengzhou University, Zhengzhou, China

**Keywords:** Internet addiction, self-efficacy, self-control, chain mediating effect, Chinese college students

## Abstract

It explores the roles of self-efficacy and self-control in physical activity and Internet addiction. And it further provides a theoretical basis for the treatment and improvement of Internet addiction among college students. This study employs the whole group sampling method. The questionnaire was conducted on 855 college students from five universities in three provinces using the Physical Activity Level Scale, the General Self-Efficacy Scale, the Self-Control Scale, and the Chinese Internet Addiction Scale (IAS). The analyses yielded three main findings. (1) A large amount of physical activity was helpful in reducing the symptoms of Internet addiction and the problematic status of each dimension among college students. (2) A large or moderate amount of physical activity was helpful in enhancing college students’ self-efficacy. Besides, a large amount of physical activity was likely to enhance college students’ self-control. (3) The condition of physical activity not only directly has the negative correlation with college students’ Internet addiction but also influences college students’ Internet addiction through two indirect ways: the mediating role of self-control and the chain mediating role of self-efficacy and self-control. These conclusions provide a deeper understanding of the protective factors of Internet addiction among Chinese college students.

## Introduction

Internet addiction refers to the phenomenon of losing control of the impulsion to use the Internet without the effect of addictive substances. The typical symptoms are excessive or poorly controlled obsessions and cravings as well as the related behaviors of impairment in academic performance, occupation and social engagement (Ndasauka et al., 2019). It is tested that the combined rate of Internet addiction among Chinese college students is 11% which is higher than some other countries. And the rate has shown a slight upward trend and gradually stabilized in the past 3 years (Shao et al., 2018). College students are the largest potential group of Internet addiction with the most serious symptoms of Internet addition, as they become less restraint after being admitted in colleges (Li et al., 2021). Although Internet addiction is not yet listed as a disease, new diagnostic criteria for Internet gaming disorder (a subtype of Internet addiction) have been incorporated into DSM-V (Diagnostic and Statistical Manual of Mental Disorders-V) and ICD -11 (The International Classification of Diseases -11), and is defined as a mental disorder, which shows the increasing international attention to Internet addiction (Ndasauka et al., 2019). The excessive use of the Internet has a negative impact on sleep (Abolghasem et al., 2016), physical health (Güzel et al., 2018), self-esteem (Budak et al., 2015), academic performance (Singh and Srivastava, 2021), and mental health (Columb et al., 2021) among college students. It has already become one of the main factors affecting the academic performance of college students (Yuanyuan et al., 2015). The current situation of Internet addiction is so grim that it has aroused widespread concern from all walks of life. Rutter (1987) proposed the theoretical model of two-factor problem behavior that the study of the factors influencing problem behavior should not only focus on the factors that produce problem behavior i.e., risk factors but also on protective factors that can directly or indirectly reduce problem behavior (Rutter, 1987). As a protective factor of Internet addiction, this study explores the psychological influence mechanism of physical activity on Internet addiction and tries to combine social control theory with the control theory of Internet addiction so as to provide theoretical and practical support for the effective prevention and control of Internet addiction among college students.

## Theories and hypotheses

### Internet addiction from the perspective of exercise psychology

From the above analysis of the psychological attribution and damage of Internet addiction, it can be seen that the psychological mechanism of Internet addiction is relatively complex, including driving factors like needs, motivation and personality trait factors like depression, loneliness, poor self-control, high feeling seeking, negative coping styles. Combining these factors to explain Internet addiction, the most representative factors are Young’s ACE model, Davis’ cognitive-behavioral model and Grohol’s stage model. Yinghai and Yujin (2009) believe that physical exercise conforms to the interpretation principles of three authoritative models of Internet addiction: ACE model, cognitive-behavior model and stage model, and can be used as an effective prevention and intervention method. The ACE model refers to the three underlying factors of availability, control and excitability to explain the formation process of compulsive Internet use. Physical exercise, as a network substitute, is closely related to personality in the process of satisfying individual needs (especially emotional needs, communication needs, self-actualization needs, etc.). Non-competitive exercise activities such as fitness and aerobic exercise have a two-way regulation function on mood, which can maintain negative mood or excessively benign mood state at the intermediate level, that is, the function of balance mechanism. Some competitive activities with outcome of winning or losing can help to improve the activation state of mood under certain conditions (Ji, 2006). Through physical exercise, individuals feel “fluid experience,” “pleasure of physical exercise,” “negative mood transfer experience,” etc., and obtain individual needs and exciting experiences (Yinghai and Yujin, 2009); in cognitive-behavioral model theory, it is believed that there are major cognitive impairments in some specific aspects, thereby aggravating the symptoms of individual Internet addiction (Yinghai and Yujin, 2009). However, numerous studies have demonstrated a moderately positive correlation between physical activity and cognitive activity (Etnier et al., 1997). When the exercise is a coordinated action that requires thinking, and when the structure and function contained in these actions are necessary to engage in certain operations, physical exercise is beneficial to cognitive activities; Grohol (1999) proposed a stage model, which considered Internet addiction. It is a staged behavior. Internet users generally go through three stages. The first stage: the novice of the Internet is fascinated by the Internet, or the experienced Internet users are fascinated by the new application software; the second stage, the user begins to avoid Internet activities that lead to addiction; the third stage: The user’s Internet activities and other activities have reached a balance. The role of physical exercise may be highlighted in the formation of a healthy sports lifestyle, or it can prevent being “fascinated” by the Internet again. Inspired by the interpretation of Grohol’s stage model, this model provides the possibility and operability of physical exercise intervention for Internet addiction tendency, Internet addiction disorder and even severe Internet addiction.

### Tool selection and symptom definition of Internet addiction

There are many tools for measuring Internet addiction, such as Young’s Internet Addiction Test, Widyanto ’s Internet Addiction Test, The Internet Addiction Scale (IAS) (Griffiths, 1998). However, considering the cultural background differences between regions and countries and the applicability to college students, this study selects the IAS compiled by Taiwanese scholar Chen et al. (2003) using college students as samples. The scale contains five common symptoms of Internet addiction, compulsiveness, withdrawal symptoms, tolerance, interpersonal health problems, and time management problems. They are used as tool-measured dimensions. Here is the introduction to the definitions of the five dimensions: (1) Obsessiveness: The individual has an inextricable desire and urge to surf the Internet (Chen et al., 2003). (2) Withdrawal symptoms: If you do not surf the Internet for a period of time, you will become obviously restless and uncontrollably want to surf the Internet, always worrying about what you are missing (Young, 1996). (3) Tolerance: Individuals need to continuously increase the time spent on the Internet to achieve their level of satisfaction (Young, 1996). (4) Interpersonal health problems: Individuals are alienated from family and friends because they indulge in the Internet for too long (Chen et al., 2003). (5) Time management problem: Individuals are delayed in their studies because they indulge in the Internet for too long (Chen et al., 2003).

### Physical activity and Internet addiction

In addition to the negative effects of Internet addiction on academic performance and psychological health, Internet addiction can largely disrupt physical activity among college students (Lepp et al., 2013). Kim et al. (2015) found that Internet addiction may negatively affect college students’ physical health by reducing the time spent on physical activity such as walking. Penglee et al. (2019) revealed that Internet addiction may be a barrier for college students to participation in physical activity and they proposed strategies to promote physical activity in higher education situations. Similarly, Barkley and Lepp (2016) stated that excessive use of the Internet among college students can be considered as a sedentary leisure behavior which leads to poor physical activity. Meanwhile, Yinghai and Yujin (2009) argued that physical activity is consistent with the explanatory principles of three authoritative models of Internet addiction, the ACE model, the cognitive-behavioral model, and the stage model. And it can be an effective method to prevent and intervene the addition. Previous studies have found that physical activity can significantly improve the symptoms of Internet addiction (Gao et al., 2012; Li et al., 2020). The results of the Meta-analysis by Jin et al. (2018) also support the above view. In clinical psychology, treatment of psychological disorders and addictive disorders, physical activity is usually used as a supplementary means by physicians or psychotherapists. However, the mechanisms through which physical activity affects college students’ Internet addiction remain to be explored. Previous empirical studies have demonstrated the predictive effect of physical activity on Internet addiction, but the current study lacks insight into the underlying psychological mechanisms of the two. To bridge this gap, we explore the effects of physical activity on Internet addiction and sought to assess the positive effects of self-efficacy and self-control on Internet addiction.

### Effect of different physical activity on Internet addiction

The current research on the effect of sports on Internet addiction is mainly intervention (Jun, 2017). A recent study extends the hierarchy to provide neurobiological and neuropsychological evidence for sports intervention on Internet addiction (Li et al., 2020), the focus of this type of research is still on whether sports are effective for Internet addiction, and no research has been conducted on the direction of what amount of exercise is most effective for Internet addiction. This study will also further verify the differences in internet addiction across three different levels of physical activity: large, medium and small.

### Self-efficacy

Self-efficacy is first proposed by the famous psychologist Bandura. It refers to people’s judgments about the ability needed to organize and perform a behavior in order to achieve a certain performance. It may affect and determine one’s choice of activities and the effort one takes in the process of performance (Bandura, 1977, 1991). Self-efficacy is the core of the social cognitive theory which Bandura proposed. The higher one’s level of self-efficacy is, the more confident he or she will be in accomplishing a behavior. In other words, he or she is more likely to perform it (Bandura, 2002). Zhengyu (2000), after analyzing the results of several studies at home and abroad, pointed out that effective participation in regular exercise can increase one’s sense of effectiveness in physical activity, improve the state of mind, and promote satisfaction with life. Therefore, there is an interaction between physical activity and self-efficacy. Heydari and Dehghanizade (2018) randomly selected 50 drug users from all drug users in Yazd City and divided them into two groups of 25 (control and experimental), the experimental group received 6 weeks of selective exercise, the control group Without any training or intervention, the results showed that the above-mentioned selective exercise had a significant effect on the improvement of self-efficacy in the experimental group (*P* < 0.05). The empirical study of Sheng et al. (2016) measured the relationship between physical exercise and self-efficacy in 1,084 middle school students, and the results showed that physical exercise was significantly positively correlated with self-efficacy. At the same time, Berte et al. (2021) verified the relationship between Internet use patterns and self-efficacy by surveying 505 Palestinian college students, and the results showed that there was a high negative correlation between Internet addiction use patterns and self-efficacy. Yang (2020) verified the effects of self-efficacy and self-control on Internet addiction among middle school students through a survey of 119 middle school students, and found that there was a significant negative correlation between self-efficacy and Internet addiction. Based on the above, hypothesis two is proposed: self-efficacy plays a mediating role in the influence of physical activity on Internet addiction among college students.

### Self-control

Self-control is the ability to suppress immediate impulses and regulate one’s behavior. Besides, it also includes the ability that individuals restrain their desires and needs and change their inherent behavioral thinking in order to live in harmony with the external environment. The ability of individuals to rationally regulate their emotions and behavior also is involved in the term self-control (Schmeichel et al., 2011). The limited self-control theory suggests that self-control requires the consumption of an individual’s resources and that lacking self-control can easily lead to addition behaviors (Baumeister et al., 2018). Previous studies have shown the positive correlation between physical activity and self-control including physical activity of different intensities (Sibley et al., 2006), different periods (Pesce et al., 2009), different types of physical activity and exercise programs (Pesce et al., 2009; Davis et al., 2011). However, Kamijo et al. (2007) noted that high-intensity physical activity may not enhance one’s self-control, which is contrary to the study of Davis et al. (2011). In addition, studies at home and abroad both have found that those with higher level of Internet addiction always have lower levels of self-control (Qifeng et al., 2013b; Akın et al., 2015). Li et al. (2020) meta-analysis of 83 primary studies with 80,681 participants determined whether students with poor self-control had greater Internet addiction, the results showed that self-control was negatively associated with Internet addiction related. Enyuan and Huiyu (2017) conducted a survey of 1,500 Chinese college students and found that self-control has a significant negative predictive effect on Internet addiction. Therefore, Hypothesis 3 is proposed: self-control plays a mediating role in the influence of physical activity on Internet addiction among college students.

### Self-efficacy and self-control

Bandura argues in his social learning theory that self-efficacy and self-control both are internal factors of the individual self. The individual factor is the driving force for the individual’s behaviors and has a direct impact on them (Bandura, 1991). Bandura holds the view that self-control is influenced by self-efficacy (Bandura, 1977). It has been noted that (Hamedani, 2013; Džinović et al., 2019) self-efficacy can directly affect self-control. The higher the self-efficacy is, the higher the self-control is. This also indicates that self-efficacy plays an important role in the construction of one’s self-control. In addition, previous studies have shown that both self-efficacy and self-control have significant negative predictive effects with Internet addiction. Ju et al. (2019) investigated 440 college students to verify the relationship between self-efficacy, self-control, and mobile phone addiction. The results showed that self-efficacy and self-control were significantly positively correlated, and indicated that self-efficacy was an indirect influence on smartphone addiction through self-control. Therefore, Hypothesis 4 was proposed: Self-efficacy and self-control play a chain mediating role between physical activity and Internet addiction.

## Current research

Previous studies exploring the relationship between physical activity and Internet addiction have mostly been intervention studies (Jun, 2017) and meta-analyses (Qiao et al., 2020). Few studies have directly explored the psychological mechanism of the impact of physical activity on Internet addiction. Despite the findings of studies showing that demographic characteristics (Farahani et al., 2018), mental illness (Farahani et al., 2018), personality disorders (Farahani et al., 2018), nostalgia (Ni et al., 2009), gender (Ni et al., 2009), neuroticism score (Tsai et al., 2009), and healthy behaviors (Tsai et al., 2009) are the influencing factors of Internet addiction. In addition, Burnay et al. (2015) used an ensemble model to collect data of 644 respondents revealed that psychological factors such as urgency, lack of perseverance, obsessive passion, and depression were all predictors of Internet addiction. However, the underlying psychological mechanisms of important psychological variables such as self-efficacy and self-control in the relationship between physical activity and Internet addiction are still unclear.

In summary, this study focused on Chinese college students and analyzed the differences between different physical activity levels on each dimensions including self-efficacy, self-control, Internet addiction. Based on this and combined with previous theories of discussing the correlation among self-efficacy, self-control, physical activity, and Internet addiction, it aims to verify the possible mechanisms of self-efficacy and self-control between physical activity and Internet addiction in order to provide the reference for reducing the Internet addiction of college students.

## Data sources and methods

### Research objects

This study employed cluster random sampling method. Based on class, it selected 950 college students (from freshman to senior) as research subjects. They come from Beijing, Henan and Inner Mongolia provinces and all study in the universities or colleges covering a number of fields and disciplines to like Beijing Normal University, Zhengzhou University, Henan College of Engineering, Zhongyuan Institute of Science and Technology, Inner Mongolia University of Science and Technology. The information related to the study subjects is detailed in Table 1. The inclusion criteria of the subjects: (1) College students (full-time only), including vocational colleges, and undergraduate colleges, while excluding college students majoring in physical education and psychology; (2) Clear consciousness. They are able to successfully complete the questionnaire without history of mental illness; (3) Voluntary participation in the investigation of this study; (4) Informed consent for the investigation work, the counselor (class teacher) issued an informed consent form, and filled out the questionnaire after signing; (5) Physical health, not included in clinical patients.

**TABLE 1:** Subjects’ information (Mean ± *SD*).

Classification	Category	Number of people	Percentage (%)	Age (years)
Total	–	855	100	19.96 ± 2.68
Gender	Male	497	58.2	20.02 ± 3.17
	Female	358	41.8	19.66 ± 1.79
Grade	Freshman year	509	59.5	18.13 ± 0.78
	Sophomore	140	16.4	18.93 ± 1.37
	Junior	149	17.5	19.67 ± 1.65
	Senior year	57	6.7	21.19 ± 1.35

### Investigation procedure

First, the staff explained the purpose and method of the study to the respondents face to face and solved their doubts in person. With the consent of the counselor (or head teacher) and the person himself or herself, the questionnaire was filled out anonymously, without involving personal information such as student number. Then, the electronic questionnaire in the form of Questionnaire Star was distributed to the participants *via* WeChat group. It took about 10 min to answer all questions. Among the 1,000 people who received electronic questionnaires, 950 people returned questionnaires (95% response rate). In order to ensure the accuracy of the data and the robustness of the structural equation model, the returned questionnaires were strictly screened to exclude invalid responses, short responses (less than 5 min), and 10 consecutive questions with the same options (each question was answered with the same answer, such as “11111111. “ or “2222222222.”), and pattern responses (e.g., “11111111.”)., pattern responses (following certain artificial rules, such as “7, 6, 5, 4, 3, 2, 1, 7, 6, 5, 4, 3, 2, 1. “ or “5, 5, 5, 4, 4, 4, 4, 3, 3, 2, 2, 2, 2, 1, 1, 1.”) to obtain a valid questionnaire. 855 valid questionnaires were obtained and the questionnaire efficiency rate was 90%. Each participant was given a small gift (such as a tip or a voucher) as a reward to thank you for participating in the survey. In accordance with local laws and institutional requirements, no ethical review or informed consent signed by the participants was required for this study. However, the process to ensure informed consent of all participants to participate was still included in the survey. When subjects accessed the electronic questionnaire, all information in the document appeared on the first page. The document stated that the survey was anonymous and that its results would be used only for scientific research without any risk to their daily lives. It also told them that participation in the study was completely voluntary. Subjects could only take the questionnaire after confirming that they had read the document and agreed to participate in the study.

### Measures

#### Physical activity level scale

The scale was compiled by the Japanese scholar Koyo Hashimoto (2005) and revised by Deqing (1994). The scale consists of 3 dimensions: intensity, duration and frequency. Each dimension has 1 item, which is scored by Likert 5 points. It follows the formula that “intensity × (time - 1) × frequency = total physical activity score.” A score of ≤ 19 indicates a small amount of physical activity, 20–42 indicates a moderate amount of physical activity, and ≥ 43 indicates a large amount of physical activity. The higher the score, the greater the amount of physical activity. The scale has high reliability and validity, with a retest reliability of 0.82. The total Cronbach’s alpha for this scale in this study was 0.774.The CFA model demonstrated a satisfactory fit [normed chi square (x^2^/df) = 4.109, comparative fit index (CFI) = 0.995, goodness of fit index (GFI) = 0.975, Tucke-Lewis index (TLI) = 0.977, root meansquare error of approximation (RMSEA) = 0.066, standardized root mean square residual (SRMR) = 0.037]. Following the recommendations of Schumacker and Lomax (2016), this scale was suitable for the sample studied.

#### General self-efficacy scale

The scale was compiled by Schwarzer et al. (1995). The Chinese version was translated and revised by Caikang et al. (2001). The scale consists of 10 items, and is scored on a 4-point Likert scale, with scores from 1 to 4 corresponding to “not at all correct” to “completely correct,” with higher scores indicating stronger general self-efficacy. The reliability and validity of the scale are high, with a retest reliability of 0.83. The total Cronbach’s alpha for this scale in this study was 0.97.The CFA model demonstrated a satisfactory fit (x^2^/df = 3.3, CFI = 0.957, GFI = 0.9, TLI = 0.945, RMSEA = 0.02, SRMR = 0.015). Following the recommendations of Schumacker and Lomax (2016), this scale was suitable for the sample studied.

#### Self-control scale

The scale was compiled and published by American scholar Tangney et al. (2004) and later revised by Shuhua and Yongyu (2008). 19 items were selected from the initial 36 items and scored on a 5-point scale, with scores ranging from 1 to 5 corresponding to “ completely inconsistent” to “ completely consistent,” among which questions 1, 5, 11, and 14 are scored positively, and the remaining items are scored backward. The higher the total score, the stronger the self-control. The scale has 5 dimensions, namely impulse control (α = 0.932), healthy habits (α = 0.927), resisting temptation (α = 0.913), focusing on work (α = 0.906), and abstaining from entertainment (α = 0.916). The scale has high reliability and validity with a retest reliability of 0.85. The total Cronbach’s alpha for this scale in this study was 0.97.The CFA model demonstrated a satisfactory fit (x^2^/df = 3.00, CFI = 0.98, GFI = 0.95, TLI = 0.976, RMSEA = 0.02, SRMR = 0.03). Following the recommendations of Schumacker and Lomax (2016), this scale was suitable for the sample studied.

#### Chinese Internet addiction scale

The scale was compiled in 1999 by Taiwanese scholars Chen et al. (2003) with college students as subjects. It has 5 dimensions including compulsiveness (α = 0.886), withdrawal symptoms (α = 0.93), tolerance (α = 0.911), interpersonal health problems (α = 0.895) and time management problems (α = 0.908), with a total of 26 question items, using Likert 4-point scale. The higher total scores indicating a higher tendency to Internet addiction. If the total score is ≥ 58, the preliminary screening is a potential Internet addict. If the total score is ≥ 68, it is diagnosed as an Internet addict. The scale has high reliability and validity with a retest reliability of 0.83. The total Cronbach’s alpha for this scale in this study was 0.976.The CFA model demonstrated a satisfactory fit (x^2^/df = 6.338, CFI = 0.924, GFI = 0.945, TLI = 0.915, RMSEA = 0.029, SRMR = 0.021). Following the recommendations of Schumacker and Lomax (2016), this scale was suitable for the sample studied.

### Statistical methods

First, descriptive statistics, reliability test, one-way ANOVA and Pearson correlation analysis were carried out on self-efficacy, self-control, physical activity and Internet addiction after normality test using SPSS 21.0. *Post hoc* multiple tests were conducted for different groups using the LSD method. Then, use Amos 24.0 software to do structural equation model and fit the chain mediation effect model to analyze the model fit. Secondly, after standardizing each research variable, the hierarchical multiple regression equation was standardized, and the equation model was analyzed by mediating effect with physical activity as the independent variable, self-efficacy and self-control as the mediating variables, and Internet addiction as the dependent variable. Incremental changes in *R*^2^ and *F* values in the results were used to assess the main effects of the study variables. Finally, after standardizing each research variable, the macro program process in SPSS was used to test the significance of the mediation effect through the bias-corrected non-parametric percentile Bootstrap method (resampled 5,000 times) (Preacher and Hayes, 2008), confidence interval No 0 is significant (*P* < 0.05; Hooper et al., 2008).

## Results

### Common method bias test

Harman’s single factor test (Podsakoff et al., 2003) was used to conduct factor analysis on all the items involved in this study. The results showed that 11 factors with eigenvalues greater than 1 were extracted by exploratory factor analysis. And the variance explained by the first factor was 20.966%, which was much lower than the critical value of 40%, which indicated that the data in this study were not affected by the common method bias.

### Differences in the effects of physical activity amount on self-efficacy, self-control, and Internet addiction

The results of the analysis showed that the main effect of physical activity amount on self-efficacy (*F* = 22.69, *p* < 0.01) and self-control (*F* = 26.55, *p* < 0.001) was statistically significant, i.e., physical activity had an effect on self-efficacy and self-control. *Post hoc* multiple comparisons revealed that college students with a large amount and moderate physical activity showed higher self-efficacy than those with a small amount of physical activity (*P* < 0.01) In terms of self-control, college students with a large amount of physical activity showed higher self-control compared to moderate physical activity and moderate physical activity compared to a small amount of physical activity (*P* < 0.01), as shown in Table 2.

**TABLE 2:** Analysis of differences in variables by physical activity groups (*M* ± *SD*).

Projects	Category	*n*	Self-efficacy	Self-control	Internet addiction	Compulsive	Withdrawal symptoms	Tolerance	Interpersonal health issues	Time management issues
Sports grade	Small	342	2.7 ± 0.65	3.05 ± 0.67	3.73 ± 0.55	3.61 ± 0.57	3.74 ± 0.56	3.75 ± 0.6	3.72 ± 0.61	3.83 ± 0.64
	Medium	274	2.93 ± 0.73	3.28 ± 0.85	3.61 ± 0.64	3.5 ± 0.69	3.62 ± 0.67	3.63 ± 0.69	3.61 ± 0.68	3.71 ± 0.71
	Big	239	3.01 ± 0.66	3.53 ± 0.82	3.32 ± 0.62	3.25 ± 0.7	3.34 ± 0.65	3.3 ± 0.67	3.31 ± 0.67	3.37 ± 0.72
Statistics analysis	*F*		22.69**	26.55**	33.95**	22.1**	29.85**	34.34**	28.86**	31.61**
	Partialη^2^		0.05	0.06	0.07	0.05	0.07	0.08	0.06	0.07
	*R* ^2^		0.05	0.06	0.07	0.05	0.06	0.07	0.06	0.06
After the fact multiple compare	LSD		Middle > Small**	Middle > Small**	Medium > Small	Medium > Small	Medium > Small	Medium > Small	Medium > Small	Medium > Small
			Big > Small**	Big > Small**	Big > Small**	Big > Small**	Big > Small**	Big > Small**	Big > Small**	Big > Small**
			Large > Medium	Large > Medium**	Large > Medium**	Large > Medium**	Large > Medium**	Large > Medium**	Large > Medium**	Large > Medium**

**p < 0.01.

The main effect of the amount of physical activity on each dimension was statistically significant with Internet addiction (*F* = 33.95, *p* < 0.01) and compulsiveness (*F* = 22.1, *p* < 0.01), withdrawal symptoms (*F* = 29.85, *p* < 0.01), tolerance (*F* = 34.34, *p* < 0.01), interpersonal health problems (*F* = 28.86, *p* < 0.01), and time management problems (*F* = 31.61, *p* < 0.01). That is to say, physical activity had an effect on Internet addiction and its other dimensions. *Post hoc* multiple comparisons revealed that a large amount of physical activity was significantly lower than individuals with moderate and small amounts of physical activity on internet addiction and its dimensions of compulsivity, withdrawal symptoms, tolerance, interpersonal health problems, and time management problems (*P* < 0.01), i.e., a large amount of physical activity compared to a small amount of physical activity and moderate college students showed less problematic behaviors for internet addiction (see Table 2).

### Analysis of the relationship among college students’ physical activity, self-efficacy, self-control and Internet addiction

The Pearson correlation analysis showed (see Table 3) that physical activity was significantly positively correlated with self-efficacy and self-control (*r* = 0.31, *P* < 0.001; *r* = –0.26, *P* < 0.001) and negatively correlated with Internet addiction and its dimensions (compulsiveness, withdrawal symptoms, tolerance, interpersonal health problems, and time management problems) (*r* = –0.27, *P* < 0.001). Self-efficacy and self-control were significantly negatively correlated with Internet addiction and its dimensions (compulsiveness, withdrawal symptoms, tolerance, interpersonal health problems, and time management problems) (*r* = –0.23, *P* < 0.001; *r* = –0.57, *P* < 0.001). Self-efficacy was significantly positively correlated with self-control (*r* = 0.42, *P* < 0.001). The significant correlations among the main variables indicated that further tests for mediating effects could be conducted (Wen et al., 2004).

**TABLE 3:** Descriptive statistics and correlation analysis of each variable.

Variables	Mean	*SD*	Physical exercise	Self-efficacy	Self-control	Internet addiction	Compulsive	Withdrawal symptoms	Tolerance	Interpersonal health issues	Time management issues
Physical exercise	3.82	0.80	1.00								
Self-efficacy	2.88	0.70	0.28**	1.00							
Self-control	3.26	0.80	0.25**	0.47**	1.00						
Internet addiction	3.58	0.62	–0.25**	–0.24**	–0.54**	1.00					
Compulsive	3.48	0.66	–0.21**	–0.23**	–0.52**	0.88**	1.00				
Withdrawal symptoms	3.59	0.64	–0.24**	–0.24**	–0.51**	0.96**	0.82**	1.00			
Tolerance	3.59	0.67	–0.24**	–0.2**	–0.48**	0.95**	0.76**	0.9**	1.00		
Interpersonal health issues	3.57	0.67	–0.23**	–0.19**	–0.49**	0.93**	0.76**	0.86**	0.9**	1.00	
Time management issues	3.66	0.71	–0.23**	–0.21**	–0.5**	0.91**	0.72**	0.85**	0.87**	0.86**	1.00

**p < 0.01.

### Relationship between physical activity and Internet addiction

This study uses Amos 24.0 to conduct structural equation model analysis to examine the mediating effect of self-efficacy and self-control between physical activity and Internet addiction. The fitting index of the model was: χ^2/^df = 3.29, *p* < 0.001, GFI = 0.91, CFI = 0.92, RMSEA = 0.05, which met the requirements of psychometrics. The equation model uses the amount of physical activity as the independent variable with self-efficacy and self-control as the mediating variable as well as Internet addiction as the dependent variable for the mediating effect analysis. The results are shown in Table 4. Physical activity positively predicted self-efficacy (β = 0.31, *P* < 0.001). Physical activity positively predicted self-control (β = 0.14, *P* < 0.001). Self-efficacy positively predicted self-control (β = 0. 38, *P* < 0.001). Physical activity negatively predicted Internet addiction (β = –0.14, *P* < 0.001). Self-control negatively predicted Internet addiction (β = –0.55, *P* < 0.001).

**TABLE 4:** Regression analysis of chain mediation model (*N* = 855).

Variables	Equation 1: (Dependent variable: Self-efficacy)			Equation 2: (Dependent variable: Self-control)			Equation 3: (Dependent variable: Internet addiction)		
			
	β	SE	*t*	β	SE	*t*	β	SE	*t*
Physical activities	0.31	0.03	8.05***	0.14	0.04	3.64***	–0.14	0.02	–3.87***
Self-efficacy				0.38	0.05	10.16***	0.04	0.03	1.24
Self-control							–0.55	0.03	–12.65***
*R* ^2^	0.10			0.2			0.34		
*F*	70.66***			135.53***			122.85***		

***p < 0.001.

**FIGURE 1:**
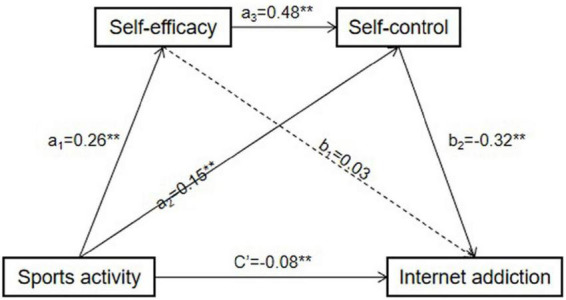
Chain mediation model of physical activity on Internet addiction. **P < 0.01.

Also, the mediation effects of each mediation path were examined by Bootstrap method (5,000 repetitions of sampling) (Wen et al., 2004). The results were showed in Table 5 and Figure 1. The 95% confidence intervals for total indirect effects, direct effects (C’), a _2_ b _2_ and a _1_ a _3_ b _2_ did not contain 0, indicating that the effects of these pathways were significant. The effect size of the direct effect path accounted for 48.26%, and the effect of physical activity on Internet addiction was significant indicating that physical activity has a direct relationship with college students’ Internet addiction. The mediating effect of self-control between physical activity and Internet addiction was significant (95%CI = –0.081, –0.030) and the proportion of effect size was 26.87%. The chain mediating effect of self-efficacy and self-control between physical activity and Internet addiction was significant (95%CI = –0.068, –0.034) and the effect size was 24.88%. The confidence interval of a _1_ b _1_ contained 0 (95%CI = –0.004, 0.027), indicating that the mediating effect of self-efficacy between physical activity and Internet addiction was not significant.

**TABLE 5:** Bootstrap analysis of the mediating effect test.

Paths	Effect	BootSE	Boot95% CI		Effect size ratio
			
			Lower limit	Upper limit	
C’	–0.097	0.023	–0.142	–0.042	48.26%
a_1_ b_1_	0.011	0.008	–0.004	0.027	–
a_2_ b_2_	–0.054	–0.013	–0.081	–0.030	26.87%
a_1_ a_3_ b_2_	–0.050	0.009	–0.068	–0.034	24.88%
Total indirect effect	–0.104	0.016	–0.125	–0.064	51.75%
Total effect	–0.201	0.0257	–0.24	–0.139	100%

C’, physical activity → Internet addiction; a1b1, physical activity → self-efficacy → internet addiction; a2b2, physical activity → self-control → Internet addiction; a1a3b2, physical activity → self-efficacy → self-control → Internet addiction; a1b1 has the opposite sign of the C’ effect size. And its proportion does not have a good effect size, so it is no longer expressed.

## Discussion

In this study, the questionnaire method was used to investigate and analyze the current situation of college students’ physical activity and Internet addiction during the normalized epidemic prevention period. And this study analyzed the influence of physical activity on Internet addiction among college students and the mediating role of self-efficacy and self-control between them.

### Differences in each variable between different physical activity amount

This study found that participation in a high level of physical activity was more likely to reduce the symptoms of Internet addiction and its dimensions (compulsivity, withdrawal symptoms, tolerance, interpersonal health problems, and time management problems) among college students than moderate and small levels of physical activity. According to the correlation analysis between physical activity and Internet addiction, it is found that physical activity significantly had a negative correlation with Internet addiction among college students, i.e., the more they participated in physical activity, the less their symptoms like compulsive, withdrawal symptoms, tolerance, interpersonal health problems, and time management problems. This result is consistent with previous studies (Gao et al., 2012; Jin et al., 2018; Li et al., 2020). Both the Internet and physical activity provide participants with a sense of interaction and entertainment. The difference between the two is that the engagement of the Internet puts one into the virtual world. While physical activity allows one to experience the real world and will guide and build the physical and mental health of the participants. One of the theoretical foundations of sports intervention therapy is that physical activity participation can occupy the time spent on the Internet, while at the same time subconsciously influencing the participants’ physiology and psychology toward positive changes. The participation of physical activity can not only effectively improve the condition of adolescent Internet addiction and the physical condition of addicts, but also contribute to promoting psychological health, exercising will and the ability of suppressing Internet addiction (Jin et al., 2018).

This study also found that the level of participation in a large amount of physical activity is more conducive to improving the self-efficacy level of college students than the level of moderate and small physical activity. In terms of self-control ability, college students with a lot of physical activity showed higher self-control ability than moderate physical activity and moderate physical activity compared with a small amount of physical activity. This is also consistent with the theoretical model of the psychological benefits of physical activity in adolescents proposed by Tomporowski et al. (2011). Based on this, the present study proved that self-efficacy and self-control, two important psychological factors, played a mediating role in the effect of physical activity on Internet addiction.

### The mediating role of self-efficacy in physical activity and college students’ Internet addiction was not significant

In this study, self-efficacy was significantly positively correlated with physical activity, and significantly negatively correlated with Internet addiction, but the mediating effect of self-efficacy between physical activity and college students’ Internet addiction was not significant, which was inconsistent with previous research theoretical implications (Heydari and Dehghanizade, 2018; Yang, 2020; Berte et al., 2021). Although numerous findings suggest a mediating role of self-efficacy between physical activity and Internet addiction, no empirical studies have directly confirmed this. As for the insignificant mediating effect of self-efficacy, our explanation is: on the one hand, the general self-efficacy scale used may refer to general and overall self-efficacy (Schwarzer et al., 1995; Caikang et al., 2001), and self-efficacy includes specific self-efficacy perspectives, such as Internet rejection self-efficacy, Internet self-efficacy, social self-efficacy, emotion regulation self-efficacy, etc. If starting from a more specific self-efficacy, the relationship may be closer. Qifeng et al. (2013a) verified the close relationship between Internet rejection self-efficacy and Internet addiction through a survey of 420 Chinese college students, Luo et al. (2010) selected 1,121 students from five universities in Wuhan to conduct research, confirmed the significant correlation and close relationship between Internet use self-efficacy and Internet control self-efficacy and Internet addiction. On the other hand, it may be found by some empirical studies that the link between self-efficacy and Internet addiction may require indirect effects of other variables (Cheng et al., 2019). And follow-up studies should verify this.

### Self-control plays a mediating role between physical activity and college students’ Internet addiction

Another important finding of this study was that self-control played a partially mediating role in physical activity on Internet addiction among college students, with an effect value of 26.87% of the total indirect effect. This supports the previous findings (Yang et al., 2019), which indicates that individuals with higher levels of self-control are able to overcome their internal desires and rationally regulate their emotions and behaviors to achieve their goals. Therefore, they can rationally control their behaviors of using the Internet and avoid forming the Internet addiction symptoms. Based on the dual system theory of self-control, self-control is composed of two systems: impulse and control. The impulse system is the process of automatic response to tempting situations such as emotions, new and different incentives and rewards. The control system, on the other hand, is a higher-order system that inhibits various impulsive responses (Schmeichel et al., 2011). People who score higher in the control system are better able to weigh and consider the consequences afterward due to their higher rational psychological quality and develop higher-order evaluation and suppression criteria when faced with temptations (Yang et al., 2019), thus suppressing the cyber-addictive behavioral impulses.

### Self-efficacy and self-control play a chain mediating role among college students’ Internet addiction

Another important finding of this study was that self-efficacy and self-control played a chain mediating role in the effect of physical activity on college students’ Internet addiction, with an effect value of 24.88% of the total indirect effect. It can be seen that physical activity promotes self-control by enhancing self-efficacy, which in turn reduces college students’ Internet addictive behaviors. Besides, the effect between self-efficacy and Internet addiction was mediated by self-control, which suggests that an increase in self-efficacy can promote a reduction in the level of Internet addiction. But this process may occur through an increase in internal self-control. Therefore, the more college students participate in physical activity, the stronger their self-efficacy and self-control subsequently increases. Therefore, regular physical activities can gradually promote the formation of a psychological positive circle, which in turn affects the improvement of Internet addiction symptoms.

### Summary

The above findings support and extend the theory of physical activity treatment for Internet addiction providing ideas for intervention correction of Internet addiction. The study also has some theoretical and practical guidance for the treatment of Internet addiction in college students, especially for the suppression of addictive behavioral impulses according to the pathway of physical activity influencing Internet addiction. Most of the research centers in the field of psychology focusing on explaining the formation mechanism of addictive behaviors but lack effective interventions and treatment methods. While the change and development of healthy behaviors can be more directly applied to individual health promotion. Aiming at the individual factors of Internet addiction, we can cultivate regular sports behavior habits to affect the enhancement of self-efficacy and self-control, thereby directly or indirectly inhibiting out-of-control behavior and reducing Internet addiction.

### Limitations

It is important to note the limitations of this study. First, this study used a cross-sectional design so it may have implications for revealing causal relationships between variables. In future studies, using a longitudinal design can help provide a developmental perspective. This study found some valuable results, but there were also certain research flaws: there was a sampling bias. The whole-group sampling method of distributing the questionnaire resulted in a biased sample of subjects in terms of gender, i.e., with more males and fewer females. This sampling bias may have some impact on the external validity of the study results. In addition, some scholars believe that addictive disorders have gender differences. In this study, only the whole group of college students was considered, and there is no separate analysis of male and female college students. Considering the representativeness of the sample in this study, future research will expand the scope of the survey to increase it to examine the relationship between physical activity and Internet addiction.

## Conclusion

A large amount of physical activity was more helpful in reducing college students’ Internet addiction symptoms and problems of each dimension. Large and moderate amounts of physical activity were more helpful in enhancing college students’ self-efficacy. And a large amount of physical activity was more likely to enhance college students’ self-control. Physical activity both directly and negatively predicted college students’ Internet addiction. Physical activity could influence college students’ Internet addiction through two indirect ways: the mediating role of self-control and the chain mediating role of self-efficacy and self-control. These findings may provide a deeper understanding of the protective factors of Internet addiction among Chinese college students.

## Data availability statement

The original contributions presented in this study are included in the article/supplementary material, further inquiries can be directed to the corresponding author/s.

## Ethics statement

Ethical review and approval was not required for the study on human participants in accordance with the local legislation and institutional requirements. The patients/participants provided their written informed consent to participate in this study.

## Author contributions

ZD conceived, designed the study, performed the data analysis, wrote the Chinese manuscript, and wrote and supplemented the English manuscript. XZ participated in the manuscript revision. ZD and XZ participated in the data collection and collation. Both authors contributed to the article and approved the submitted version.
